# Dietary Protein and Blood Pressure: A Systematic Review

**DOI:** 10.1371/journal.pone.0012102

**Published:** 2010-08-11

**Authors:** Wieke Altorf – van der Kuil, Mariëlle F. Engberink, Elizabeth J. Brink, Marleen A. van Baak, Stephan J. L. Bakker, Gerjan Navis, Pieter van 't Veer, Johanna M. Geleijnse

**Affiliations:** 1 Top Institute Food and Nutrition, Wageningen, The Netherlands; 2 Division of Human Nutrition, Wageningen University, Wageningen, The Netherlands; 3 Human Studies Group, TNO Quality of Life, Zeist, The Netherlands; 4 Department of Human Biology, NUTRIM School for Nutrition, Toxicology and Metabolism, Faculty of Health, Medicine and Life Sciences, Maastricht University, Maastricht, The Netherlands; 5 Kidney Center, University Medical Center Groningen and University of Groningen, Groningen, The Netherlands; Lerner Research Institute, United States of America

## Abstract

**Background:**

Elevated blood pressure (BP), which is a major risk factor for cardiovascular disease, is highly prevalent worldwide. Recently, interest has grown in the role of dietary protein in human BP. We performed a systematic review of all published scientific literature on dietary protein, including protein from various sources, in relation to human BP.

**Methodology/Principal Findings:**

We performed a MEDLINE search and a manual search to identify English language studies on the association between protein and blood pressure, published before June 2010. A total of 46 papers met the inclusion criteria. Most observational studies showed no association or an inverse association between total dietary protein and BP or incident hypertension. Results of biomarker studies and randomized controlled trials indicated a beneficial effect of protein on BP. This beneficial effect may be mainly driven by plant protein, according to results in observational studies. Data on protein from specific sources (e.g. from fish, dairy, grain, soy, and nut) were scarce. There was some evidence that BP in people with elevated BP and/or older age could be more sensitive to dietary protein.

**Conclusions/Significance:**

In conclusion, evidence suggests a small beneficial effect of protein on BP, especially for plant protein. A blood pressure lowering effect of protein may have important public health implications. However, this warrants further investigation in randomized controlled trials. Furthermore, more data are needed on protein from specific sources in relation to BP, and on the protein-BP relation in population subgroups.

## Introduction

Elevated blood pressure (BP) is an independent risk factor for cardiovascular diseases (CVD) and renal impairment.[1] There is no evidence for a threshold effect: from systolic BP levels as low as 115 mmHg onward, risk of CVD doubles for each increment of 20 mmHg.[1] It has been estimated that, at population level, a reduction in systolic BP of only 2 mmHg would result in a 6% reduction in fatal stroke, and a 4% reduction fatal coronary heart disease (CHD).[2]

Well-known dietary and lifestyle interventions to prevent hypertension include moderate physical activity, maintenance of normal body weight, low alcohol and salt intake, and a diet rich in fruits, vegetables, and low-fat dairy products.[2], [3] More recently, interest has grown into dietary patterns and macronutrient intakes, including protein.[4], [5] Whether protein content of the diet or type of protein is important for human BP is, however, unclear. We systematically reviewed all scientific literature, published before June 2010, on dietary protein in relation to human BP, with a focus on specific types of protein and possible interactions with age, gender, BP level, and overweight.

## Methods

Ethical approval was not required for this review because only published data were included.

### Search strategy

A systematic search was performed in MEDLINE (www.ucbi.ulm.nih.gov) to identify studies on the association between dietary protein and BP, published before June 2010. Search terms on dietary protein and BP or hypertension were used to search for words in title or abstract and Medical Subject Headings. The search was limited to studies in human adults and English-language literature. In addition, we performed a manual search using reference lists of original articles and previous reviews [6]–[9]. For all studies, we retrieved the original publication.

We selected any observational study or trial that examined the relationship between dietary protein and BP in humans. All titles, abstracts, and full papers of potentially relevant studies were assessed for eligibility based on predefined inclusion and exclusion criteria. Papers were excluded: 1) if data on exposure (dietary protein) or outcome (BP, hypertension) was not reported, 2) if no data were reported on the relationship between exposure and outcome, 3) if the exclusive effect of protein could not be calculated (e.g. BP studies that focused on dietary patterns, or soy combined with isoflavones). Furthermore, review papers were excluded, as were drug trials and studies conducted in patient groups or pregnant women.

### Data collection and data synthesis

From each included paper we extracted data on protein intake, source of protein, and BP values or estimated risk of hypertension according to a predefined standard form. In addition, we extracted data on design, place of study, number of participants, population characteristics (including initial BP, sex, and age), dietary assessment method (food frequency questionnaire (FFQ), 24-hour recall, food diary, biomarker), adjustment for confounders, and measures of variation.

To allow better comparison of results from observational studies we expressed associations in these studies by standard units of protein intake that correspond to approximately 1 SD of protein intake in the Dutch population, i.e. 25 g/d (3.5 en%) for total protein, 11 g/d (1.4 en%) for plant protein, and 23 g/d (2.9 en%) for animal protein.[10], [11]

## Results

**Figure 1:**
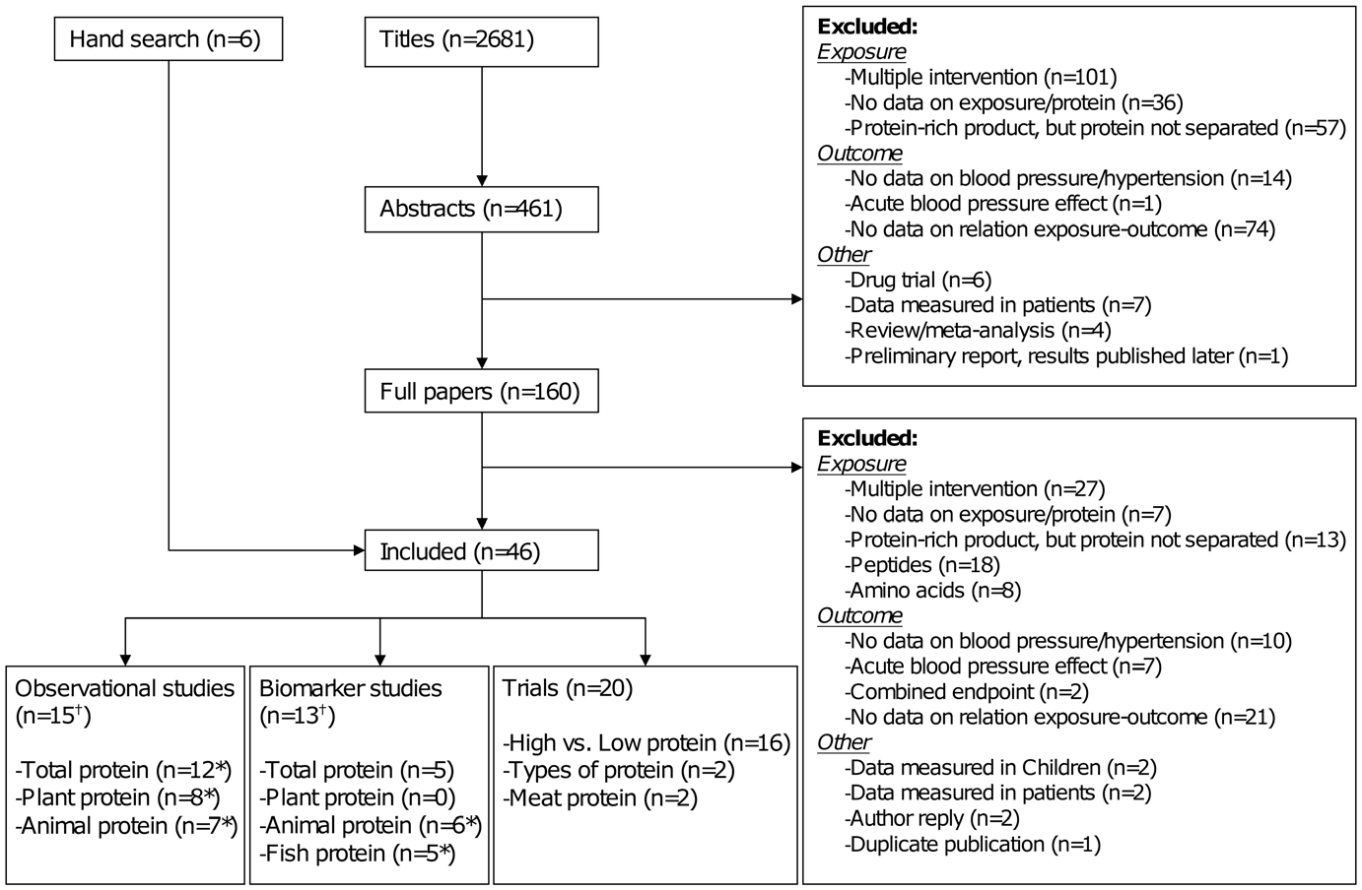
Flow chart of systematic literature search. *Numbers overlap because several studies investigated different types of protein. ^†^Numbers overlap because two studies investigated protein intake using questionnaires as well as biomarkers.

The systematic search in MEDLINE resulted in 2,681 titles to be screened. Inclusion criteria were fulfilled by 40 papers, and the hand search yielded another 6 papers (**Figure 1**). In total, 15 observational studies, 13 biomarker studies and 20 trials were selected.

### Total dietary protein and BP: observational data

Twelve observational studies focused on habitual total protein intake and BP or risk of hypertension (**table 1**). Most of these studies had a cross-sectional design and showed predominantly weak inverse associations.[12]–[20] However, although hypothesis-generating, a major drawback of a cross-sectional design is that protein intake and BP are assessed at the same moment in time, which makes it difficult to address the temporality of the association. Subjects with elevated BP, or otherwise at increased cardiovascular risk, may have changed their food intake (including protein intake) upon medical advice. Causality can, therefore, be better established in prospective studies.

**Table 1:** Observational studies of *total protein intake* and blood pressure.

Author, year	respondents	Initial BP (mmHg)	Habitual protein intake	Dietary assess-ment	BP outcome (SBP/DBP)	BP outcome per 25 g/d or 3.5 en% (SBP/DBP)	P-value	Statistical adjustment
**Cross-sectional studies**								
Pellum, 1983[16]	61 normotensive US adults mean age ∼24	M: 119/73F: 107/68	M: 101 g/d; F: 65 g/d (≈14 en%)	3-d food record	−0.13/… mmHg per g/d	∼ −3.33/… mmHg per 25 g/d	…/…	Sex, serum HDL, exercise, fat intake
Havlik, 1990[13]	402 male US twins aged 42–56 y	128/82	15 en% ( = 75 g/d)	FFQ	…/+0.11 mmHg per g/d of energy adjusted protein	…/∼ +2.75 mmHg per 25 g/d	NS/0.02	Weight, serum cholesterol, triglycerides, total energy intake
Wang, 2008[20] (PRE-MIER)	810 untreated pre- or mild hypertensives aged 25–79 y	135/85	16 en%	2×24 h recall	−0.08/+0.03 mmHg per en%	∼ −0.28/∼ +0.11 mmHg per 3.5 en%	0.41/0.73	Age, sex, race, weight, waist, exercise, education, income, antihypertensive drugs, study site, baseline BP, alcohol, energy intake, intake of Ca and K, urinary creatinine, urinary Na
He, 1995[14]	827 Chinese adults mean age ∼38^1^	∼113/70	∼12 en% (≈93 g/d)	3×24-h recall	−3.6/−2.2 mmHg per SD ( = 39 g/d)	∼ −2.28/∼ −1.38 mmHg per 25 g/d	<0.05/NS	Age, BMI, alcohol, urinary Na, energy intake, resident area
Reed, 1985[17]	6496 Japanese men in Hawaii aged 46–69 y	…	…	24-h recall	−3 mmHg/−1 mmHg for Q5 (≥122) vs. Q1 (<67 g/d)	∼ −0.85/∼ −0.28 mmHg per 25 g/d	<0.001/0.03	Age
Umesawa, 2009[19]	7585 Japanese men and women aged 40–69 y^1^	M: 137/83; F: 135/81	M: 83 g/d;F: 65 g/d	Single 24 h recall	−0.29/−0.42 mmHg per 25.5 g/d	∼ −0.28/∼ −0.41 mmHg per 25 g/d	NS/<0.05	age, gender, BMI, smoking, alcohol, community, use of antihypertensive medication, intake of sodium, potassium, and calcium
					M: +0.07/−0.31 mmHg per 27.4 g/d	M: ∼ +0.06/∼ −0.28 mmHg per 25 g/d	<0.01/NS	age, gender, BMI, smoking, alcohol, community, use of antihypertensive medication, intake of sodium, potassium, and calcium
					F: −0.61/−0.55 mmHg per 20.6 g/d	F: ∼ −0.74/∼ −0.66 mmHg per 25 g/d	NS/<0.05	age, gender, BMI, smoking, alcohol, community, use of antihypertensive medication, intake of sodium, potassium, and calcium
Masala, 2008[15] (EPIC)	7601 Italian women aged 35–64 y	123/79	…	FFQ	+0.38/+0.60 mmHg per log(g/d)	∼ +1.22/∼ +1.93 mmHg per 25 g/d	0.76/0.43	Age, BMI, waist circumference, smoking, education, physical activity, energy intake
Garcia-Palmieri, 1984[12]	7932 men from Puerto Rico aged 45–64 y	…	…	24-h recall	SBP: between -0.03 and +0.03 mmHg per g/d depending on subgroup (urban/rural, middle-aged/old age); DBP: …	∼+0.13/… mmHg per 25 g/d	NS/…	Age, smoking, weight, education, serum glucose, heart rate, intake of milk, fat, carbohydrates, coffee, alcohol
Stamler, 1996b[18] (MRFIT)	11342 US men aged 35–57 y^1^	125/84	17 en%	4–5×24-h recall	−0.06/−0.06 mmHg per en%	∼ −0.20/∼ −0.21 mmHg per 3.5 en%	<0.01/<0.001	Age, race, BMI, education, smoking, serum cholesterol, antihypertensive drugs, Na and K intake, alcohol and caffeine intake; corrected for regression dilution bias
**Prospective studies**								
Stamler, 2002[21]	1714 men, aged 40–55 y^1^	135/87	15 en%	FFQ	+0.05/−0.02 mmHg per year per en%	∼ +0.16/∼ −0.05 mmHg per 3.5 en%	0.04/0.16	Age, height, weight (+ change), education, alcohol, smoking
Liu, 1996[22] (CARDIA)	4146 US blacks and whites aged 18–30 y^1^	∼110/69	∼15 en%	FFQ	∼ −0.16/∼ −0.34 mmHg per year per 3 en%	∼ −0.20/∼ −0.40 mmHg per year per 3.5 en%	NS/NS	Age, BMI, education, exercise, smoking, alcohol, hostility score, use of antihypertensive medication, intake of K and Ca
Alonso, 2006[23] (SUN)	5880 Hispanic, university graduates, mean age ∼36 y	…	∼18 en%	FFQ	HR (95%-CI) = 0.9 (0.6; 1.4) for Q5 vs.Q1 of energy adjusted protein	NA	0.51	Age, sex
					Multivariable HR (95%-CI) = 0.8 (0.4; 1.4) for Q5 vs. Q1 of energy adjusted protein		0.26	Age, sex, BMI, exercise, alcohol, smoking, hypercholesterolemia, intake of total energy, Na, fruit, vegetables, fiber, caffeine, magnesium, potassium, low-fat dairy, MUFA, SFA

BP = blood pressure, SBP = systolic blood pressure, DBP = diastolic blood pressure, M = men, F = women, en% = energy percentage; 95%-CI = 95% confidence interval, MUFA = monounsaturated fat, PUFA = polyunsaturated fat, SFA = saturated fat, Na = sodium, K = potassium, Ca = calcium, BMI = body mass index; NS = not statistically significant (p>0.05); …  =  value not given.

1 Users of anti-hypertensive medication were not excluded.

So far, only three studies prospectively examined the association of total dietary protein with change in BP or incident hypertension. Total protein intake was not clearly associated with change in systolic BP after 8 years of follow up in 1714 US men (+0.16 mmHg per y per 3.5 en% systolic, p = 0.04) [21], and after 7 years of follow up in 4146 young US adults (−0.20 mmHg per year per 3.5 en% systolic, p>0.05) [22]. It should be noted that in these two studies respondents using antihypertensive medication were not excluded from the analyses, which may have affected the associations. In 5880 university graduates of the prospective SUN cohort, not using antihypertensive medication, a non-significant 20% lower 2-year hypertension risk was found (p = 0.26).[23] In this study the population was quite young (mean age ∼36 y), and BP may not have been as sensitive to influence from protein intake as in an older population.

Concluding, most cross-sectional studies on total protein intake and BP or incident hypertension showed a weak inverse association, whereas no clear conclusion could be drawn from prospective studies. A small beneficial effect on BP may exist, but well conducted prospective studies and randomized controlled trials may provide better estimates of a protein effect on BP.

### Biomarkers of total dietary protein and BP: observational data

Daily urinary nitrogen excretion, about 85% excreted in the form of urea, correlates with dietary protein as calculated from weighed food records (r = 0.4–0.8) and reflects ∼80% of total protein intake.[24] As shown in **table 2**, in five cross-sectional studies urinary total nitrogen [25] or urinary urea nitrogen [11], [25]–[28] was used to estimate the association between total protein intake and BP.

**Table 2:** Observational studies of *biomarkers of total protein intake* and blood pressure.

Author, year	respondents	Initial BP (mmHg)	Habitual protein intake	Dietary assessment	BP outcome (SBP/DBP)	P-value	tatistical adjustment
**cross-sectional studies**							
Kihara, 1984[28]	1120 traditional Japanese aged over 30 y	M: 132/79	…	Urea nitrogen/Cr in single-spot urine (mol:mol)	M: +0.13/+0.02 mmHg per unit (partial regression coefficients)	<0.05/NS	…
		F: 129/76	…	Urea nitrogen/Cr in single-spot urine (mol:mol)	F: −0.04/−0.01 mmHg per unit (partial regression coefficients)	NS/NS	…
Iseki, 2003[27]	1299 Japanese adults, mean age ∼49 y^1^	∼121/74	∼1.1 g/kg/day	Urea nitrogen in single spot urine	−3.0/−2.4 mmHg per g/kg/day	…/…	Unadjusted
Cirillo, 2002[26]	3705 Italian adults aged 25–74 y^1^	127/76	…	Urea nitrogen in overnight urine	−5.2/… mmHg per log(urea) in mmol/h	<0.01/…	Age, sex, BMI, exercise, alcohol, smoking, antihypertensive drugs, urinary Na, K, Ca, creatinine clearance
Elliott, 2006[11] (INTER-MAP)	4680 respondents from China, Japan, UK and USA aged 40–59 y^1^	119/74	China: 12 en%; Other countries: 15–16 en%	Urea nitrogen in 24 h urine	M: −0.77/−0.40 mmHg per 5.34 g/24 h (2 SD)	NS/NS	…
					F: −1.11/−0.41 mmHg per 5.34 g/24 h	NS/NS	
Stamler, 1996a[25] (INTER-SALT)	10020 adults from 32 countries worldwide aged 20–59 y^1^	119/73	…	Total nitrogen in 24-h urine	−0.50 mmHg per g/−0.41 mmHg per g	<0.01/<0.01	Age, sex, BMI, alcohol and 24 h urinary Na, K, Ca, Mg; corrected for regression dilution bias
					Older respondents (40–59 y): −0.92/−0.48 mmHg per g	<0.01/<0.05	Age, sex, BMI, alcohol and 24 h urinary Na, K, Ca, Mg; corrected for regression dilution bias
					Younger respondents (20–39 y): −0.20/−0.38 mmHg per g	…/<0.05	Age, sex, BMI, alcohol and 24 h urinary Na, K, Ca, Mg; corrected for regression dilution bias
Stamler, 1996a (INTER-SALT)	10020 adults from 2 countries worldwide aged 20–59 y^1^	119/73	…	Urea nitrogen in 24-h urine	−0.57/−0.50 mmHg per g	<0.05/<0.01	Age, sex, BMI, alcohol and 24 h urinary Na, K, Ca, Mg; corrected for regression dilution bias

BP = blood pressure, SBP = systolic blood pressure, DBP = diastolic blood pressure, M = men, F = women, Na = sodium, K = potassium, Ca = calcium, BMI = body mass index; PUFA = polyunsaturated fat, SFA = saturated fat, 3MH = 3-methylhistidine; Cr = creatinine; NS = not statistically significant (p>0.05); …  =  value not given.

1 Users of anti-hypertensive medication were not excluded.

In the large INTERSALT-study, including 10,020 adults from 32 countries, an inverse association of −0.5 mmHg systolic (p<0.01) per g of total 24-h urinary nitrogen was observed.[25] Also in 4,680 respondents from the INTERMAP study, 24 h urea nitrogen was inversely related to systolic BP (−0.9 mmHg per 5.34 g), although this was not statistically significant.[11] In the remaining studies, summarized in table 2, single spot or overnight urines were used to estimate protein intake.[26]–[28] Although these estimates are less reliable than estimates from 24-h urine, the results were in line with those of the studies mentioned above.

Concluding, in studies among participants that are in nitrogen balance, good agreement has been found between one or two 24-h urine collections and diet-history estimates of protein intake.[24] Findings from biomarker studies, therefore, suggest that protein intake may have a beneficial effect on BP.

### Total dietary protein and BP: trial data

In 16 trials the BP effect of a high protein diet was assessed (**table 3**). Most trials were only small (number of participants per intervention group: n = 7 to n = 30), and the conflicting results may be due to chance findings.[29]–[39] In one of the larger trials, a parallel trial in which 121 type 2 diabetes patients received counseling on normal or reduced protein intake, an increase in BP was found (+5.4 mmHg systolic, p = 0.07).[40] However, the low range of intake may have influenced the results. Another large parallel trial among 311 obese women, in which different weight loss diets were compared, showed a decrease in systolic BP of −5.7 mmHg systolic (p value not given).[41] However, contrast in protein intake was low (2.3 en%), and BP decrease may be a result of exchange in carbohydrates and fat instead of increase in protein intake. Other large studies showed a decrease in BP on a high protein diet, although no clear dose-response relation could be distinguished.[5], [42], [43] In 100 obese participants with metabolic syndrome, systolic BP changed −6 mmHg (p<0.05) with 6 en% higher protein intake [42], and in 141 obese adults 6 en% higher protein intake resulted in a BP change of −4.6 mmHg (p = 0.04) [43].

**Table 3:** Trials of *total protein intake* and blood pressure.

Author, year	Blin-ding	Participants	Initial BP (mmHg, Interven-tion vs. control)	Type of intervention	Intake of protein in control group	ΔProtein	ΔCH	ΔFat	Duration of interven-tion	ΔSBP/ΔDBP due to intervention	P-value
**Cross over trials**											
DeHaven, 1980[32]	…	7 healthy obese participants, aged 23–38 y	114/69	Pure prot ( = boiled turkey), low caloric (400 Kcal) diet vs. mixed (turkey+grapejuice) low caloric diet	50 en%	“High”	“High”	0	3 to 5.5 weeks for each diet	+5/+1 mmHg*	…
Daniels, 1990[31]	…	7 normotensive healthy adults (6 M, 1 F), aged 22–49 y	…./…	High prot vs. low prot diet	0.55 g/kg/d	+1.45 g/kg/d	“high”	“similar”	4 days	+3/+2 mmHg	NS/NS
Papakon-stantinou, 2010[38]	sb	17 obese, newly diagnosed type 2 diabetes patients, aged 30–65 y	134/86 vs. 134/80	Low caloric (−700 kcal) high protein low fat diet vs. low caloric low protein high fat diet.	61 g/d ≈15 en%	+56 g/d ≈+15 en%	−4 g/d ≈0 en%	−28 g/d ≈−15 en%	4 weeks per diet, 3 weeks wash out	−9/−5 mmHg	<0.001/<0.001
Sacks, 1984[39]	sb	23 US vegans, aged 22–41 y	112/74	High prot supplement (60 g wheat prot: 40 g soy protein) vs. low prot supplement (rice prot)	70 g	M: +60 g/d; F: +45 g/d	M: +2 g/d; F: +1 g/d	…	6 weeks for each diet	+1/+0.6 mmHg*	NS/NS
Appel, 2005[5] (Omni−Heart)	db	164 US participants (55% African Americans), mean age 64 y	131/77	Prot rich diet (∼50% plant prot) vs. CH rich diet	15 en%	+10 en%	−10 en%	0	6 weeks foreach diet	−1.4/−1.2 mmHg	0.002/<0.001
										In prehypertensives:−0.9/−0.9 mmHg	0.047/0.01
										In hypertensives: −3.5/ −2.4 mmHg	0.006/0.008
Appel, 2005 (Omni-Heart)	db	164 US participants (55% African Americans), mean age 54 y	131/77	Prot rich diet (∼50% plant prot) vs. fat rich diet	15 en%	+10 en%	0	−10 en%	6 weeks for each diet	−0.1/−0.4 mmHg	0.90/0.20
										In prehypertensives: 0.0/−0.4 mmHg	0.98/0.27
										In hypertensives: −0.2/−0.5 mmHg	0.79/0.51
**parallel trials**											
Ferrara, 2006[33]	db	15 healthy men in exercise training project, aged 18–36	111/72 vs. 110/76	High vs. normal prot diet	15 en%	+7 en%	−10 en%	+3 en%	6 months	−2.1/+0.9 mmHg	NS/NS
Hendler, 1988[34]	…	17 healthy obese participants, mean age ∼31	120/79 vs. 121/79	Pure prot, low caloric (440 kcal) diet vs. mixed low caloric diet	41 en%	+54 en%	−53 en%	−1 en%	3 weeks	−2/−8 mmHg	NS/NS
Meckling, 2007[37]	…	30 overweight/obese women (premenopausal), aged 20–62	134/82 vs. 129/82	High prot low caloric (1383 kcal) diet vs. control low caloric (1391 kcal) diet	∼16 en%	+8.2 en%	−12.9 en%	+4.8 en%	12 weeks	−3/−4 mmHg*	…/…
Meckling, 2007	…	30 overweight/obese women (premenopausal), aged 20–62	134/82 vs. 129/82	High prot low caloric (1217 kcal) diet+ exercise vs. control low caloric (1260) diet + exercise	∼18 en%	+19.0 en%	−14.6 en%	−3.3 en%	12 weeks	0/0 mmHg*	…/…
Burke, 2001[30]	o	41 Australian treated hypertensives, mean age ∼57 y^1^	133/75	Soy prot suppl vs. maltodextrin supplement (2×2 RCT with soluble fiber)	12 en%	+11 en%	−13en%	+2 en%	8 weeks	−5.9 mmHg/−2.6 mmHg	<0.01/<0.01
Leidy, 2007[36]	o	46 obese women (8 drop-outs), aged 28–80	109/69 vs. 113/73	High protein (pork) low caloric (750 kcal) vs. normal protein (milk) low-caloric diet	18 en%	+12 en%	−12 en%	0	12 weeks	−2/+2 mmHg	NS/NS
Hodgson, 2006[35]	o	60 Australian participants, mean age 57 y^1^	129/80 vs. 134/77	CH replaced by lean red meat prot vs. maintaining normal diet	18.6 en%	+5.2 en%	−4.3 en%	−0.6 en%	8 weeks	−4.0/−1.3 mmHg	0.02/0.25
Brinkworth, 2004[29]	…	64 obese type 2 diabetes patients, mean age ∼62 y^1^	148/83 vs. 140/76	High vs. low prot diet; Both groups 8 weeks energy restricted (∼6.7 MJ/day) and 4 weeks energy balance	15 en%	+15 en%	−15 en%	0	12 weeks	−0.3/−1.7 mmHg	NS/NS
Muzio, 2007[42]	sb	100 obese participants with MetS, mean age ∼52 y^1^	142/85 vs. 141/82	Low vs. high CH diet. Both diets providing a deficit of ∼500 kcal	13 en%	+6 en%	−17 en%	+11 en%	5 months	−6/−1 mmHg*	<0.05/…
Pijls, 1999[40]	sb	121 type 2 diabetes patients, mean age ∼63 y^1^	138/79 vs. 138/79	Counseling by dietician; reduced SFA alone vs. reduced SFA + reduced prot (isocaloric)	0.95 g/kg/d	+0.19 g/kg	+1 g/d	SFA: +2.9 g/d; UFA: +5 g/d	6 months	+5.4/+4.6 mmHg	0.07/<0.01
Delbridge, 2009[43]	…	141 obese (≥27 kg/m^2^) men and women aged 18–75 y	135/85 vs. 131/83	High protein diet vs. high CH diet after 12 weeks of low caloric diet (≈500−550 kcal/d)	22 en%*	+6 en%*	−12 en%*	+2 en%*	12 months	−4.6/−1.1 mmHg	0.04/0.58
Gardner, 2007[41]	sb	311 obese women (premenopausal), aged 25–50 y	116/75	Comparison of several weight loss diets; Atkins (AT)vs. Zone (ZO)	18 en%	0.6 en%	−10.9 en%	9.8 en%	12 months	−4.3/−2.3 mmHg	…/…
				Atkins vs. LEARN (LE)	18.5 en%	2.5 en%	−12.7 en%	11.4 en%	12 months	−4.5/−2.2 mmHg	…/…
				Atkins vs. Ornish (OR)	18.3 en%	+2.3 en%	−17.9 en%	+14.5 en%	12 months	−5.7/−3.7 mmHg	…/…

o = open; sb = single-blind; db = double-blind; MetS = metabolic syndrome; SBP = systolic blood pressure, DBP = diastolic blood pressure, M = men, F = women, en% = energy percentage; CH = carbohydrates; prot = protein; SFA = saturated fat, UFA = unsaturated fat; NS = not statistically significant (p>0.05); …  =  value not given; *Best guess on basis of graph/implicit data in paper.

1 Users of anti-hypertensive medication were not excluded.

In almost all trials the high protein diet was compared with a high carbohydrate diet. The only study in which two different control diets were included was the OmniHeart trial.[5] In this 6-week, fully controlled cross-over feeding trial in 164 healthy US adults partial substitution of carbohydrates (10 en%) with protein significantly lowered systolic BP with −1.4 mmHg systolic (p = 0.002). No difference in BP response was observed when the protein-rich diet was compared with a diet high in mono-unsaturated fat (−0.1 mmHg systolic, p = 0.90). Recently, a trial was conducted in which only a high fat diet was included as control diet.[38] In this trial, however, the number of participants was very low (n = 17), and the systolic BP effect of −9 mmHg may be a chance finding.

In conclusion, the results of trials suggest that increased intake of protein may be beneficial to BP, although no clear dose – response association could be distinguished. From the results of the OmniHeart study, the only trial in which two different isocaloric control diets (high in carbohydrates and high in fat) were used, a conclusion can be drawn that both protein and mono-unsaturated fat have blood pressure lowering properties. However, it is also possible that a reduced intake of carbohydrates, rather than a higher intake of mono-unsaturated fat or protein, is responsible for a reduced blood pressure. In a trial on macronutrients and blood pressure it is important to keep energy intake in both treatment groups constant, to rule out blood pressure effects of energy and change in weight. Measurements of blood pressure effects after high intake of one of the macronutrients, therefore, will always be relative to the intake of the other two macronutrients, and the answer to the question whether total protein intake itself influences blood pressure may never be given, unless specific mechanisms are found through which protein intake may affect blood pressure.

### Dietary plant protein and BP: observational data

The association between dietary plant protein and BP or hypertension was examined in 8 observational studies (**Table 4**). Most cross-sectional studies showed an inverse association [11], [14], [15], [19], [20], [44], and this was confirmed in prospective studies [20], [21], [23]. In a prospective study among 1714 men a systolic BP difference of −0.34 mmHg per year per 1.4 en% (p<0.01) was found after a follow-up of 8 y.[21] It should be noted, however, that estimates were not adjusted for important potential confounders like sodium and potassium. In two other studies, in which estimates were adjusted for these confounders, a 21% reduction in hypertension risk per en% of plant protein intake (p = 0.08) was found after 18 months of follow-up in 810 untreated pre- or mild hypertensives of the PREMIER study [20], and a 50% lower 2 year hypertension risk for the highest quintile of plant protein intake versus the lowest quintile (p = 0.06) was found in 5880 university graduates of the SUN cohort [23].

**Table 4:** Observational studies of *plant protein intake* and blood pressure.

Author, year	respondents	Initial BP (mmHg)	Habitual plant protein intake	Dietary assess-ment	BP outcome (SBP/DBP)	BP outcome per 11 g/d or 1.4 en%	P-value	Statistical adjustment
**Cross-sectional studies**								
Joffres, 1987[44] (Honolulu heart study)	615 men of Japanese ancestry	∼…/…	…	24 h recall	−4.6/−1.8 mmHg for Q4 (36–78 g/d) vs. Q1(4–21 g/d)	∼ −1.14/∼ −0.44 per 11 g/d	0.006/0.02	Age, BMI
Wang, 2008[20] (PRE-MIER)	810 untreated pre- or mild hypertensives aged 25–79 y	135/85	5 en%	2×24 h recall	−0.98/−0.70 mmHg per en%	∼ −1.37/∼ −0.98 mmHg per 1.4 en%	<0.01/<0.01	Age, sex, race, weight, waist, exercise, education, income, antihypertensive drugs, study site, baseline BP, alcohol, energy intake, intake of Ca and K, urinary creatinine, urinary Na
He, 1995[14]	827 Chinese adults mean age ∼38^1^	∼113/70	∼9 en% (≈76 g/d)	3×24-h recall	−1.6/−1.3 mmHg per SD ( = 44 g/d)	∼ −0.41/∼ −0.32 mmHg per 11 g/d	NS/NS	age, BMI, alcohol, urinary Na, energy intake, residential area
Elliott, 2006[11] (INTER-MAP)	4680 respondents from China, Japan, UK and USA aged 40–59 y^1^	119/74	China: 10 en% Other countries: 5–7 en%	4×24 h recall	−1.11/−0.71 mmHg per 2.8 en% (2 SD)		<0.01/<0.05	Age, sex, weight, height, exercise, alcohol, sample, history CVD or DM, family history of hypertension, special diet, supplement use, 24 h urinary Na, K
					−1.01/−0.95 mmHg per 2.8 en% (2 SD)	∼ −0.51/∼ −0.48 mmHg per 1.4 en%	NS/<0.05	Additionally adjusted for: intake of Ca, SFA, PUFA, cholesterol, fiber
Umesawa, 2009[19]	7585 Japanese men and women aged 40–69 y^1^	M: 137/83; F: 135/81	M: 40 g/d; F: 31 g/d	Single 24 h recall	+0.59/ −0.31 mmHg per 13.1 g/d	∼ +0.50/ ∼ −0.26 mmHg per 11 g/d	<0.05/ NS	age, gender, BMI, smoking, alcohol, community, use of antihypertensive medication, intake of sodium, potassium, and calcium
					M: +0.64/−0.26 mmHg per 14.0 g/d	M: ∼ +0.50/∼ −0.20 mmHg per 11 g/d	NS/NS	age, gender, BMI, smoking, alcohol, community, use of antihypertensive medication, intake of sodium, potassium, and calcium
					F: +0.46/−0.41 mmHg per 10.8 g/d	F: ∼ +0.47/∼ −0.41 mmHg per 11 g/d	NS/<0.05	age, gender, BMI, smoking, alcohol, community, use of antihypertensive medication, intake of sodium, potassium, and calcium
Masala, 2008[15] (EPIC)	7601 Italian women aged 35–64 y	123/79	…	FFQ	+1.18/ −0.23 mmHg per log(g/d)	∼ +3.79/ ∼ −0.74 mmHg per 11 g/d	0.28/ 0.73	Age, BMI, waist circumference, smoking, education, physical activity, energy intake and intake of animal protein
**Prospective studies**								
Wa/ng 2008, (PRE-MIER)	810 untreated pre- or mild hypertensives aged 25–79 y	135/85	5 en%	2×24 h recall	Change from baseline to 6 months: −0.53/−0.37 mmHg per en%	∼ −0.74/∼ −0.52 mmHg per 1.4 en% per 6 months	0.08/0.09	Age, sex, race, weight, waist, exercise, education, income, antihypertensive drugs, study site, baseline BP, alcohol, intake of Ca and K, urinary creatinine, urinary Na + 6-month changes in several variables
Wang 2008, (PRE-MIER)	810 untreated pre- or mild hypertensives aged 25–79 y	135/85	5 en%	2×24 h recall	OR (95%-CI) for hypertension = 0.79 (0.60–1.02) per en%	NA	0.08	Age, sex, race, weight, waist, exercise, education, income, antihypertensive drugs, study site, baseline BP, alcohol, intake of Ca and K, urinary creatinine, urinary Na
Stamler, 2002[21]	1714 men aged 40–55^1^	135/87	3.5 en%	FFQ	−0.24/−0.14 mmHg per year per en%	∼ −0.34/∼ −0.19 per 1.4 en%	<0.01/<0.01	Age, height, weight (+ change), education, alcohol, smoking
Alonso, 2006[23] (SUN)	5880 Hispanic, university graduates, mean age ∼36 y	…	…	FFQ	HR (95%-CI) = 0.8 (0.5; 1.2) for Q5 vs. Q1 of energy adjusted protein intake	NA	0.46	Age, sex
					Multivariable HR (95%-CI) = 0.5 (0.2; 0.9) for Q5 vs. Q1 of energy adjusted protein intake	NA	0.06	Age, sex, BMI, exercise, alcohol, smoking, hypercholesterolemia, and intake of total energy, Na, fiber, caffeine, magnesium, potassium, low-fat dairy, MUFA, SFA

BP = blood pressure, SBP = systolic blood pressure, DBP = diastolic blood pressure, M = men, F = women, en% = energy percentage; 95%-CI = 95% confidence interval, MUFA = monounsaturated fat, PUFA = polyunsaturated fat, SFA = saturated fat, Na = sodium, K = potassium, Ca = calcium, BMI = body mass index; NS = not statistically significant (p>0.05); … = value not given.

1 Users of anti-hypertensive medication were not excluded.

In conclusion, results from observational studies indicate an inverse association between dietary plant protein and BP. However, despite adjustment for many potential confounders in multivariable models, residual confounding (e.g. by other macronutrients, fiber or flavonoid intake) in observational studies cannot fully be excluded.

### Dietary animal protein and BP: observational data

In 7 observational studies the relationship between dietary animal protein and BP was investigated (**Table 5**), with results from cross-sectional studies being inconclusive [11], [15], [19], [20], [45]. In studies with a prospective design no association or only weak associations were observed, with systolic BP differences of −0.06 mmHg per 2.9 en% (p = 0.84) after 6 months in 810 untreated pre- or mild hypertensives [20], and +0.16 mmHg per 2.9 en% per year (p<0.01) in 1714 men.[21] Furthermore, no difference in hypertension risk with high intake of animal protein was observed in 5880 university graduates of the SUN cohort.[23]

**Table 5:** Observational studies of *animal protein intake* and blood pressure.

Author, year	respondents	Initial BP (mmHg)	Habitual animal protein intake	Dietary assess-ment	BP outcome (SBP/DBP)	Standardized BP outcome per 23 g/d or 2.9 en% (SBP/DBP)	P-value	Statistical adjustment
**Cross-sectional studies**								
Zhou, 1994[45]	705 rural Chinese aged 45–59 y^1^	∼117/75	0.1 to 5.3 en%	24 h recall	Inverse association (only standardized regression coefficients presented in the paper)	…	…	Age, BMI, heart rate, alcohol
Wang, 2008[20] (PRE-MIER)	810 untreated pre- or mild hypertensives aged 25–79 y	135/85	11 en%	2×24 h recall	+0.08/−0.03 mmHg per en%	∼ +0.23/∼ −0.09 mmHg per 2.9 en%	0.42/0.71	Age, sex, race, weight, waist, exercise, education, income, antihypertensive drugs, study site, BP, alcohol, energy intake, intake of Ca and K, urinary creatinine, urinary Na
Elliott 2006[11] (INTER-MAP)	4680 respondents from China, Japan, UK and USA aged 40–59 y^1^	119/74	China: 2.5 en% Other countries: 9–10 en%	4×24 h recall	+0.20/−0.02 mmHg per 5.8 en% (2 SD)		NS/NS	Age, sex, weight, height, exercise, alcohol, sample, history CVD or DM, family history of hypertension, special diet, supplement use, 24 h urinary Na, K
					+0.22/+0.25 mmHg per 5.8 en% (2 SD)	∼ +0.11/∼ +0.12 mmHg per 2.9 en%	NS/NS	Additionally adjusted for: intake of Ca, SFA, PUFA, cholesterol, fiber
Umesa-wa, 2009 [19]	7585 Japanese men and women aged 40–69 y^1^	M: 137/83; F: 135/81	M: 43 g/d; F: 35 g/d	Single 24 h recall	−0.56/−0.17 mmHg per 19.9 g/d	∼ −0.64/∼ −0.20 mmHg per 23 g/d	<0.05/NS	age, gender, BMI, smoking, alcohol, community, use of antihypertensive medication, intake of sodium, potassium, and calcium
					M: −0.29/−0.11 mmHg per 22.3 g/d	M: ∼ −0.30/∼ −0.11 mmHg per 23 g/d	NS/NS	
					F: −0.80/−0.23 mmHg per 16.4 g/d	F: ∼ −1.12/∼ −0.32 mmHg per 23 g/d	<0.001/NS	
Masala, 2008 [15] (EPIC)	7601 Italian women aged 35–64 y	123/79	…	FFQ	+0.99/+0.58 mmHg per log(g/d)	∼ +3.18/∼ +1.87 mmHgper 23 g/d	0.21/0.23	Age, BMI, waist circumference, smoking, education, physical activity, energy intake and intake of plant protein
**Prospective studies**								
Wang 2008 (PREMIER)	810 untreated pre- or mild hypertensives aged 25–79 y	135/85	11 en%	2×24 h recall	Change from baseline to 6 months: −0.02/+0.01 mmHg per en%	∼ −0.06/∼ +0.03 mmHg per 2.9 en% per 6 months	0.84/0.97	Age, sex, race, weight, waist, exercise, education,income, antihypertensive drugs, study site, baseline BP, alcohol, intake of Ca and K, urinary creatinine, urinary Na + 6-month changes in several variables
Wang 2008 (PREMIER)	810 untreated pre- or mild hypertensives aged 25–79 y	135/85	11 en%	2×24 h recall	OR (95%-CI) for hypertension = 0.99 (0.93–1.07) per en%	NA	0.90	Age, sex, race, weight, waist, exercise, education, income, antihypertensive drugs, study site, baseline BP, alcohol, intake of Ca and K, urinary creatinine, urinary Na
Stamler 2002[21]	1714 men, aged 40–55^1^	135/87	11.5 en%	FFQ	+0.06/−0.002 mmHg per year per en%	∼ +0.16/∼ −0.01 mmHg per 2.9 en%	<0.01/0.44	Age, height, weight (+ change), education, alcohol, smoking
Alonso 2006[23] (SUN)	5880 Hispanic, university graduates, mean age ∼36 y	…	…	FFQ	HR for hypertension (95%-CI) = 1.1 (0.7; 1.6) for Q5 vs. Q1 of energy adjusted protein intake	NA	0.84	Age, sex
					Multivariate HR (95%-CI) = 1.0 (0.6; 1.8)		0.70	Age, sex, BMI, exercise, alcohol, smoking, hypercholesterolemia, and intake of total energy, Na, fruit, vegetables, fiber, caffeine, magnesium, potassium, low-fat dairy, MUFA, SFA

BP = blood pressure; SBP = systolic blood pressure; DBP = diastolic blood pressure; en% = energy percentage; 95%-CI = 95% confidence interval; MUFA = monounsaturated fat; PUFA = polyunsaturated fat; SFA = saturated fat; Na = sodium; K = potassium; Ca = calcium; BMI = body mass index; CVD = cardiovascular disease; DM = diabetes mellitus; Q = quintile; NS = not statistically significant (p>0.05); …  =  value not given.

1 Users of anti-hypertensive medication were not excluded.

In conclusion, observational studies provide no evidence for an association of animal protein with BP. However, also for these studies, despite inclusion of many potential confounders in their multivariate model, residual confounding (e.g. by intake of other macronutrients or salt) cannot be excluded.

### Biomarkers of dietary plant protein or animal protein and BP: observational data

We did not find any studies that used a biomarker specifically for plant protein intake. With regard to animal protein intake, urinary excretion of 3-methylhistidine (3-MH) has been suggested as marker of meat consumption because it is synthesized in the muscle of mammals and released and excreted in urine after intake of muscle protein.[46] Six cross-sectional studies included in this review used urinary 3-MH excretion to estimate animal protein intake in predominantly Asian populations (**Table 6**). Overlap between studies may exist, since all populations formed part of the study population of the World Health Organization Cardiovascular Disease and Alimentary Comparison (CARDIAC) study, which is an international population-based cross-sectional study in more than 20 countries, among which are China and Japan. All studies showed inverse associations with BP. However, because studies were conducted mainly in Asian populations, results may not be generalizable to other populations. Furthermore, urinary 3-MH may partly reflect muscle catabolism in the human body itself, i.e. during starvation, cachexia, or heavy physical activity.[52] This phenomenon was not taken into account in the various studies, and overestimation of associations between animal protein and BP could have occurred. The findings of these biomarker studies, therefore, should not be overemphasized. A challenge for future protein research will be to find reliable biomarkers for plant and animal protein and intake of protein from specific dietary sources.

**Table 6:** Observational studies of *biomarkers of animal protein intake* and blood pressure.

Author, year	Respondents	Initial BP (mmHg)	Initial biomarker level	Biomarker assessment	BP outcome (SBP/DBP)	P-value	Statistical adjustment
**Cross-sectional studies**							
Liu, 2000b[78] (CARDIAC)	619 Chinese subjects aged 48–56 y	120/70	3MH = 211 µmol/d	24 h urinary 3MH	−3.25/−2.86 mmHg/88 µmol/d	<0.01/<0.01	Age, sex, BMI, alcohol urinary Na, urinary K
					BMI<26 kg/m^2^ (n = 497): −2.39/−2.24 mmHg/88 µmol/d	<0.01/<0.01	
					BMI≥26 kg/m^2^ (n = 30): −6.75/−4.82 mmHg/88 µmol/d	0.03/0.04	
Zhou, 1994[45]	705 rural Chinese aged 45–55 y	∼115/73	…	Overnight urinary 1MH	Inverse association with DBP (standardized regression coefficients)	…	Age, BMI, heart rate, ethnic groep
Liu, 2002[48] (CARDIAC)	1135 Chinese subjects aged 48–56 y	122/73	3MH = 198 µmol/d	24 h urinary 3MH	−0.02/−0.02 mmHg/µmol/d	0.048/0.01	Age, sex, BMI, urinary Na/K, urinary Ca, urinary Mg
Liu, 2002 (CARDIAC)	1135 Chinese subjects aged 48–56 y	122/73	3MH:Cr = 191 µmol/mg	24 h urinary 3MH:Cr (µmol/mg)	−0.02/−0.02 mmHg/unit	0.02/0.01	Age, sex, BMI, urinary Na/K, urinary Ca, urinary Mg
Liu, 2000a (CARDIAC)	1151 Chinese subjects aged 48–56 y	120/71	3MH:Cr = 216 µmol/g	24 h urinary 3MH:Cr ratio (µmol/g)	−0.046/−0.039 mmHg/unit	0.001/ <0.001	Age, sex
Liu, 2001[79] (CARDIAC)	1614 Chinese subjects from 4 different ethnic groups, aged 48–56 y	∼129/79	3MH:Cr = 142 to 258 µmol/mg	24 h urinary 3MH:Cr ratio (µmol/mg)	−0.04 to −0.25/ −0.10 to −0.36 (Partial correlation coefficients)	<0.01/ <0.01	Age, sex, urinary Na
Liu, 2000a[80] (CARDIAC)/	1681 Japanese subjects aged 48–56 y	124/75	3MH:Cr = 206 (µmol/g)	24 h urinary 3MH:Cr ratio (µmol/g)	…/−0.008 mmHg/unit	NS/0.012	Age, sex
Liu, 2002[48] (CARDIAC)	1991 Chinese subjects aged 48–56 y	123/73	…	24 h urinary 3MH	OR for hypertension(95%-CI) = 0.60 (0.40; 0.90) for ≥253 vs. <253 µmol/d	0.01	Age, sex, BMI, urinary Na/K, urinary Ca, urinary Mg
Liu, 2002 (CARDIAC)	1991 Chinese subjects aged 48–56 y	122/73	…	24 h urinary 3MH:Cr (µmol/mg)	OR for hypertension(95%-CI) = 0.38 (0.24; 0.59) for ratio ≥224 vs. <224	<0.01	Age, sex, BMI, urinary Na/K, urinary Ca, urinary Mg
Yamori, 1990[51] (CARDIAC)	7334 subjects from 20 different countries aged 50–54 y	…	…	24 h urinary 3MH:Cr ratio (mol/mol)	−568/−339 mmHg/unit	<0.05/<0.05	Unadjusted

cs = cross-sectional; BP = blood pressure; SBP = systolic blood pressure; DBP = diastolic blood pressure; Na = sodium; K = potassium; Ca = calcium; Mg = magnesium; BMI = body mass index; 1MH = 1-methylhistidine; 3MH = 3-methylhistidine; Cr = creatinine; NS = not statistically significant (p>0.05).

**Table 7:** Trials on *intake of types of protein* and blood pressure.

Author, year	Study design	Participants	Initial BP (plant protein intervention vs. animal protein intervention)	Type of intervention	ΔProtein	ΔCH	ΔFat	Duration of intervention	ΔBP due to intervention (SBP/DBP)	P-value
Wheeler, 2002[53]	x, …	23 type 2 diabetes patients, with albuminuria^1^	151/85 mmHg	Meals with only plant prot vs. meals with 60% nimal and 40% plant prot	0	0	0	6 weeks for each diet	+1/+1 mmHg*	0.90/0.75
Brussaard, 1981[54]	p, …	49 healthy normotensive students	123/69 mmHg vs. 124/69 mmHg	Soy prot isolate vs. casein prot isolate (2:1)	0	0	0	4 weeks	+0.6/+0.3 mmHg*	NS/NS

x = cross-over, p = parallel; SBP = systolic blood pressure, DBP = diastolic blood pressure, M = men, F = women, en% = energy percentage; CH = carbohydrates; prot = protein; NS = not statistically significant (p>0.05); …  =  value not given; *Best guess based on graph/implicit data in paper.

1 Users of anti-hypertensive medication were not excluded.

### Dietary plant protein or animal protein and BP: trial data

The BP response after protein intake from plant and animal sources was investigated in only 2 randomized controlled trials (**table 7**). A systolic BP effect of +1 mmHg systolic (p = 0.90) was seen in 23 type 2 diabetics after a diet containing protein only from plant sources (from soy, vegetables, and legumes) compared to a diet in which 60% of the plant protein was replaced by animal protein (from beef, poultry, fish, and milk).[53] However, the number of 23 participants is low, and this BP effect was not significant. Furthermore, these participants suffered from albuminuria, which may have influenced the results on BP. In 49 healthy students a soy protein isolate resulted in a non significant systolic BP response of +0.6 mmHg (p-value unknown) compared to a casein protein isolate.[54] However, because in this trial only soy protein and casein protein were investigated, we cannot extrapolate these findings to plant protein and animal protein from a mix of sources.

In summary, only 2 small trials evaluated the BP effect of plant protein versus animal protein. More evidence on the BP effect of plant and animal protein is needed from large randomized controlled BP trials.

### Dietary protein from specific sources and BP

Only few observational studies addressed the relation of protein from specific sources (e.g. fish, meat) to BP. In five studies the association with BP was examined for urinary taurine [49], [50], [55] or serum taurine [45], [56] which the authors regarded as a biomarker of seafood protein intake (data not in table). Three of these studies were conducted among Asian populations (n = 705 to n = 1,681) [45], [49], [50], whereas the others were conducted in Brazil (n = 57) and USA (n = 168).[55], [56] In all these studies inverse associations with BP were observed, but no information about the strength of the associations was given.

The BP effect of meat protein was only investigated in two trials (data not in table).[57], [58] In a parallel trial among 64 hospital staff members, a diet with 40% of protein from meat sources (from beef, chicken, lamb, sausage, pork, and prawns) resulted in a non-significant BP effect of −1.8 mmHg systolic and −1.2 mmHg diastolic (p-value not given) compared with a diet in which the meat protein was replaced by plant protein (from cereals, vegetables, legumes, and nuts).[57] In a small cross-over trial among 35 men no difference in BP effect was seen (no p-value given) between a diet including 50% of protein from meat (from pork, beef, and chicken) compared with a diet in which the meat protein was replaced by non-meat protein (from vegetables, eggs, and dairy).[58]

Because isoflavones may influence BP [59], several studies on soy could not be taken into account because observational data were not adjusted for isoflavone intake [60]–[64], or because, in trials, soy protein contained isoflavones [65]–[69]. To the best of our knowledge, there are at present no other studies on specific protein sources and BP. Epidemiological studies and randomized controlled trials in this field are, therefore, warranted.

### Dietary protein and BP in subgroups of the population

In several studies specific subgroup analyses were conducted to identify subgroups whose BP is more sensitive for protein intake. We explored, furthermore, whether differences in protein-BP associations could be identified in the results of studies among specific populations.

In the OmniHeart trial the effect of total dietary protein was more pronounced in hypertensives than in prehypertensives (−3.5 mmHg versus −0.9 mmHg for systolic BP).[5] This difference of protein effect in subgroups of BP could not be recognized in observational studies. In trials, however, populations with, on average, elevated BP were more sensitive to the BP lowering effect of protein than populations with, on average, normal BP (Out of 9 trials in populations with elevated BP [5], [29], [30], [35], [37], [38], [40], [42], [43] 7 trials showed a decrease in BP with high protein intake [5], [30], [35], [37], [38], [42], [43], whereas out of 7 trials in populations with normal BP [31]–[34], [36], [39], [41] only 2 trials [34], [41] showed a decrease).

With regard to age, in the INTERSALT study a stronger inverse association of urinary nitrogen with BP was observed in respondents aged 40–59 y than in respondents aged 20–39 y (systolic BP: −0.9 mmHg/g versus −0.2 g/d).[25] Furthermore, inverse associations were found more often in studies conducted in participants aged over 50 (out of 5 studies [5], [29], [30], [35], [40], in 3 studies an inverse association or a BP lowering effect was found [5], [30], [35]) than in studies conducted in younger participants (out of 9 studies [16], [22], [23], [31]–[34], [39], [41], in 4 studies an inverse association was found [16], [23], [34], [41]). However, the number of studies that were conducted among these specific populations was small, and solid conclusions cannot be drawn.

In a study on urinary 3-MH and BP, the inverse association was more pronounced in respondents with a BMI higher than 26 kg/m^2^ than in respondents with a normal BMI (Δ systolic BP = −6.8 mmHg versus −2.39 mmHg per 88 µmol urinary 3-MH/d).[47] Among the other studies, however, only one study was explicitly conducted among normal weight respondents[14], so no conclusion can be drawn on difference in sensitivity related to weight, although studies in overweight/obese participants often showed inverse associations (Out of 11 studies [5], [18], [20], [29], [32], [34]–[37], [41], [42], 7 studies showed an inverse association or a decrease in BP with high protein intake [5], [29], [34], [35], [37], [41], [42]).

Finally, in two studies subgroup-analyses were conducted for men and women, but no effect modification was shown.[19], [28] Also in studies that were specifically conducted in men [12], [13], [17], [21], [25], [33] or women [36], [37], [41], no difference in sensitivity was seen.

In conclusion, the possible beneficial effect of protein intake on BP seems stronger in people with higher initial BP and, possibly, in older people. Additional predefined subgroup analyses in future epidemiologic studies and trials in which subgroups are compared, may provide better insight into the role of dietary protein in BP.

## Discussion

A reduction in systolic BP of only 2 mmHg may already result in a 6% reduction in fatal stroke, and a 4% reduction fatal coronary heart disease (CHD).[2] Knowledge on the effect of dietary protein, therefore, may have an important public health impact. A substantial body of evidence suggests a, possibly weak, beneficial effect of total dietary protein on BP, which may be most apparent in populations with elevated BP and possibly older populations. We cannot exclude, however, that this effect is due to a lower carbohydrate intake. In observational studies more often an inverse association was found for plant protein than for animal protein. The beneficial effect of protein, therefore, may be mainly due to protein from plant sources. Data on protein from specific sources are too scarce to draw any conclusions.

The aim of the current systematic review was to give a comprehensive overview of the evidence on dietary protein and human BP, published until June 2010. Papers were independently screened by 2 reviewers, and data of 46 studies were extracted using a predefined procedure. Several other reviews on protein and BP have already been conducted in the past.[6]–[9] However, the most comprehensive review of these is already 14 years old.[9] Furthermore, the present review is the first to focus on possible BP effects of different protein types and on sensitivity of population subgroups.

Several methodological issues of studies need to be addressed. First, in observational studies, even after extensive adjustment for potential confounders, residual confounding may exist from other nutrients associated with protein intake, or from energy, which is not only correlated to protein, but also to several other BP-determinants like exercise, BMI, and dietary pattern. It is difficult to say how much the remaining confounding from known or unknown nutrients that are correlated to plant or animal protein, have influenced the estimates in observational studies. Randomized controlled trials in which the effects of plant protein and animal protein are compared, keeping other nutrients constant, are needed. Second, a diet high in one type of protein (animal protein or plant protein) does not necessarily mean that the other protein type is replaced, as a diet may be high or low in both types of protein. Most of the observational studies investigating types of protein did not adjust their estimates for intakes of other protein types. In randomized trials these factors are more standardized.[70] Third, respondents in observational studies may be misclassified according to their self-reported protein intake, which may dilute the protein-BP association.[71] Fourth, for investigation of long-term effects of protein on BP, an observational study is the most suitable type of study, because of the costs of a trial. However, contrasts between high and low protein intake are often larger in trials than in observational studies. Short term effects of protein on BP can, therefore, be more easily detected in trials. Finally, all observational studies were conducted in the general population, whereas trials were more often conducted in selected populations that are possibly more sensitive to BP interventions. However, in several trials BP was the secondary outcome [29], [31]–[34], [36], [37], [40]–[42], [53]. If participants in these studies were not blinded for the results of the BP-measurements, bias may have been introduced, because awareness of BP may influence participants' lifestyle or other behavior.

The underlying mechanism for a potential beneficial effect of protein on BP has not yet been clarified. Several hypotheses have been put forward. First, dietary protein has been related to synthesis of cellular ion channels, which may indirectly influence the pathways in BP regulation.[25] High protein intake may induce natriuresis, leading to lower BP.[26], [65], [72] Second, experiments suggest that dietary protein or protein fractions could improve insulin sensitivity and thereby BP.[73]–[75] Third, dietary protein supplementation may result in a higher concentration of the amino acids tyrosine and tryptophan in regions of the brain or blood vessel wall, triggering a vasodilatory response.[76] The amino acid arginine, which is a substrate for nitric oxide, may play a role in vasodilatation, although it is unclear whether dietary intake of arginine is relevant in this respect.[75], [77] Finally, as has already been stated in this review we cannot exclude that a lower BP is related to a lower carbohydrate intake instead of a higher protein intake.

In conclusion, evidence suggests a small beneficial effect of protein on BP, especially for plant protein. More data on protein from specific sources like dairy, grain or nuts and data in population subgroups should be obtained from epidemiological studies. Furthermore, there is a need for BP trials that focus on plant and animal protein and protein from specific sources. Preferably, these trials should be conducted in untreated (pre)hypertensive people. Finally, studies aimed at potential BP lowering mechanisms related to protein intake are warranted.
